# Mean Platelet Volume (MPV) as an indicator of  disease activity and severity in lupus

**DOI:** 10.12688/f1000research.10763.3

**Published:** 2017-03-16

**Authors:** Abidullah Khan, Iqbal Haider, Maimoona Ayub, Salman Khan

**Affiliations:** 1 Department of Medicine, Khyber Teaching Hospital, Peshawar, 25000, Pakistan

**Keywords:** systemic lupus erythematosus, blood platelets, platelets aggregation

## Abstract

**Background: **Amongst the different clinical and laboratory parameters used to monitor disease activity in systemic lupus erythematosus (SLE), mean platelet volume (MPV) is a novel biomarker. Although MPV has been studied in other rheumatological conditions like rheumatoid arthritis, its role in adult SLE needs to be defined, especially in Pakistan. **Methods:** The aim of this study was to evaluate the role of MPV as a biomarker of disease activity in SLE. Fifty patients were recruited through a consecutive non-probability sampling technique for this cross-sectional study.  On the basis of their SLE disease activity index (SLEDAI) score of greater or lesser than 5, these 50 participants were divided into two equal groups respectively;25 patients with active SLE, and another 25 participants with stable, inactive lupus. MPV was measured in each group and compared using SPSS version 16. MPV was also correlated with SLEDAI and erythrocyte sedimentation rate (ESR). Independent sample t-test and Spearman’s rho and Pearson’s correlation tests were applied. Sensitivity and specificity of MPV were checked through ROC analysis. **Results: **The MPV of patients with active SLE (n=25, mean [M]=7.12, SD=1.01) was numerically lower than those in the inactive-SLE group (n=25, M= 10.12, SD=0.97), and this was statistically significant ( *P*<0.001). MPV had an inverse relationship with both ESR (r=-0.93, *P*<0.001) and SLEDAI (r _s_= -0.89, *P*<0.001). However, there was a strong positive correlation between ESR and SLEDAI (r _s_=0.90, *P*<0.001). For MPV, a cutoff value of less than 8.5fl had a sensitivity of 92% and a specificity of 100% ( *P*< 0.001). **Conclusions:** Higher disease activity in SLE is associated with a correspondingly low MPV.

## Abbreviations

MPV: Mean Platelet Volume, SLEDAI: Systemic Lupus Erythematosus Disease Activity Index, SLE: Systemic Lupus Erythematosus, ESR: Erythrocyte Sedimentation Rate, CRP: C-reactive Protein, ACR: American College of Rheumatology.

## Introduction

Systemic lupus erythematosus (SLE) is a chronic autoimmune disorder that can affect any organ system of the body. It has an annual incidence of 5 per 100,000 of the general population ^1^. There are racial and ethnic variations, with higher rates reported in Black and Hispanic peoples ^2^. Usually, this disease with protean manifestations has a remitting relapsing course; however, it has a tendency to vary from acutely progressive to chronic indolent forms ^1, 2^.

The clinical manifestations of SLE range from constitutional symptoms, such as fever, sweats, weight loss, joint pains and skin rashes (including the classic butter fly rash), to more serious features, including the involvement of the central nervous system and kidneys. However, to make a clinical diagnosis of SLE, simultaneous or sequential presence of 4 out of a total of 11 criteria, proposed by the American College of Rheumatology (ACR), must be present ^3, 4^.

Considering the remitting relapsing nature of most cases of SLE, it is important to have a biomarker to monitor its disease activity. Although, the most effective and reliable tool to measure SLE disease activity is still open to debate, there are fortunately many validated measures, including the Systemic Lupus Activity Measure, Systemic Lupus Erythematosus Disease Activity Index (SLEDAI), Lupus Activity Index, European Consensus Lupus Activity Measurement, and British Isles Lupus Activity Group ^5^. These tools have been found to be beneficial in day to day practice ^6, 7^.

Notable issues, apart from some other technical limitations, with the aforementioned severity assessment indices are that, these validated instruments are confusing, lengthy and time consuming. However, very recently, mean platelet volume (MPV) has been shown to be a very good and easily accessible marker of disease activity in lupus as well as in antiphospholipid syndrome (APS) ^8– 10^. Moreover, a recent study not only demonstrated an inverse relationship between MPV and disease severity in lupus, but also suggested serum albumin as another effective indicator of prognosis in such people ^11^. Although MPV has been studied well as a simple but reliable inflammatory biomarker in several diseases, such as rheumatoid arthritis, scleroderma, rheumatic fever, ankylosing spondylitis and even chronic obstructive pulmonary disease, there is still a relative scarcity of its role as a disease severity indicator in lupus ^12– 16^. Therefore, we performed the present study to find out whether MPV does or does not correlate with SLEDAI and whether it can be used a predictor of lupus severity and activity.

## Methods

### Patient characteristics

This cross-sectional study was conducted in the Department of Medicine of Khyber Teaching Hospital (KTH; Peshawar, Pakistan) between January 2015 and July 2016. Medical records, intranet of our hospital and referrals by the general practitioners were the sources of recruitment. Patient information sheet, letters and direct contact by the investigators, who were directly involved in the provision of healthcare to potential subjects, were the chief methods of recruitment. This study was approved by the Ethics Review Committee of the hospital and a written informed consent was obtained from every participant (approval number, KTH/2015/Med-A/86C). The patient sample was collected using a consecutive-non-probability sampling technique. Nevertheless, as is true of cross-sectional studies, confounding and sample selection bias may be limitations to the generalization of our results.

Patients from both genders in the age range of 18–70 years, and those with both either newly diagnosed or pre-existing SLE were included in the study. In order to avoid bias, only those patients with a normal platelet count were included. This is because, MPV is influenced by the number of platelets in circulation. The ACR criteria for the diagnosis of SLE was used as a diagnostic tool.

Individuals who had history of smoking, acute or chronic infectious diseases, hemoglobin >16.5 g/dl, thrombocytopenia (platelets <150,000/mm ^3^), hypertension, angina pectoris, myocardial infarction, diabetes mellitus, hypo- or hyperthyroidism, anti-phospholipid syndrome, recurrent miscarriage, amyloidosis, thrombosis and acute or chronic renal failure were excluded from the study. Patients who had either clinical, biochemical or serological evidence of an autoimmune disorder other than SLE, such as rheumatoid arthritis, Sjogren’s syndrome or scleroderma, were also excluded from the study. All these aforementioned conditions influence platelet number and/or size. As these conditions act as potential confounders, their inclusion would have caused a selection bias in our study results.

The sample size was calculated by using the frequency of low MPV in patients with actively flaring lupus from the previous studies, along-with a 5% margin of error and 95% CI on the WHO’s formula for determination of sample size in health studies ( http://www.who.int/chp/steps/resources/sampling/en/). Moreover, based on recent published data regarding the association of MPV with disease activity in SLE ^9– 11^, sample size was doubly checked by using comparisons of means. Sample size calculation was performed by using *α* = 0.05, *β* = 0.20, and two tails. It was demonstrated that, a sample size of 50 (25 participants per group) would yield a statistically significant study with a power of 80%. A total of 64 patients were assessed initially. However, only 50 of them satisfied the inclusion and exclusion criteria. The 50 participants recruited were divided into two equal groups, 25 subjects each in the active-SLE and the inactive-SLE groups, as detailed below.

### SLEDAI

The participants were divided into two groups, active and inactive SLE groups. The division of the patients into two groups was based on their final score from using the Systemic Lupus Erythematosus Disease Activity Index-2000 (SLEDAI-2000) ^17^. The previously published literature demonstrated a mean cut-off score of 5 or higher on SLEDAI-2000 as an effective indicator of actively flaring lupus ^17^. Considering a mean cut-off score of 5 or more on SLEDAI-2000 as significant indicator of disease activity in lupus, those who scored 5 or higher were classified as active-SLE, while those with a final mean score of less than 5 were regarded as patients with inactive-SLE. Patients with active-SLE, fulfilling the inclusion criteria (SLEDAI-2000), were admitted to any one of the five medical wards of KTH for further workup and treatment. However, those with stable inactive disease were recruited in the study from the Outpatient Department of KTH.

### Measurement of MPV

A total of 5ml of venous blood was taken in an EDTA tube from every participant for the measurement of complete blood count, including hemoglobin, white blood cells, platelets, MPV, and erythrocyte sedimentation rate (ESR). All the blood samples were analyzed within less than one hour after sampling. The complete blood count, including all the hematological parameters, was performed using the same hematology analyzer, Medonic. The tests were performed and read by the same laboratory technician of KTH.

### Data analysis

All the data was entered on a structured questionnaire specifically designed for this study ( Supplementary File 1). Data was transferred to and analyzed using SPSS version 16. Means and standard deviations were determined for quantitative variables. Independent sample t-test was run to compare means of MPV between the two groups. ROC analysis was performed to estimate cutoff values for sensitivity and specificity of MPV. Spearman’s rho correlation test was used to assess any association of MPV and ESR with SLEDAI. Similarly, MPV was correlated with ESR through Pearson’s correlation test. *P* value of less than 0.05 was considered as significant.

## Results

Of the 50 participants, 84% were female and 16% were male. There were 4 males and 21 females in each of the active- and inactive-SLE groups, respectively. Other demographic details are shown below ( Table 1). The overall mean age of all the participants was 27.94±2.52 years. The mean age of the patients in the active-SLE group (M=27.84, SD=2.06) was comparable to the inactive-SLE group (M=29.60, SD= 2.38). The clinical features of patients with active- and inactive-SLE are shown in Table 2 and Table 3, respectively. In the active-SLE group, 11 (44%) patients had evidence of clinically significant proteinuria; details of the histological sub-type of lupus nephritis are given in Table 4.

**Table 1.** Demographic details of patients in each group (n=25).

Characteristic	Active-SLE (n=25)	Inactive-SLE (n=25)
Males	16%	16%
Females	84%	84%
Pregnant Females	16%	24%
Outdoor workers (sun exposed)	28%	24%
Indoor workers (least sun exposed)	72%	76%
History of recurrence	20%	36%
Pakistanis	84%	88%
Afghans	16%	12%

**Table 2.** Overview of clinical characteristics of patients with active-SLE.

CLINICAL FEATURES	PATIENTS, N (%)
Polyarthritis	22 (88)
Butterfly rash	16 (64)
Photosensitivity	20 (80)
Patchy alopecia	15 (60)
Pleurisy	14 (56)
Pericarditis	2 (8)
Neuropsychiatric problems	13 (52)
Oral ulcers	20 (80)
Proteinuria (>0.5gm/day)	11 (44)
Anti-dsDNA antibodies	24 (96)
Antinuclear factor	23 (92)

**Table 3.** Overview of the clinical characteristics of patients with inactive-SLE.

CLINICAL FEATURES	PATIENTS, N (%)
Non-specific aches and pains	17 (67)
Malaise	14 (54)
Dyspepsia	13 (53)
Anorexia	10 (40)
Photosensitivity	6 (23)
Non-scarring alopecia	5 (19)

**Table 4.** Overview of the renal histology in 11 patients with active-SLE and proteinuira.

Histological type of lupus nephritis	Patients, N (%)
Focal proliferative glomerulonephritis	3 (27)
Diffuse proliferative glomerulonephritis	7 (64)
Membranous glomerulonephritis	1 (9)

**Figure 1.**
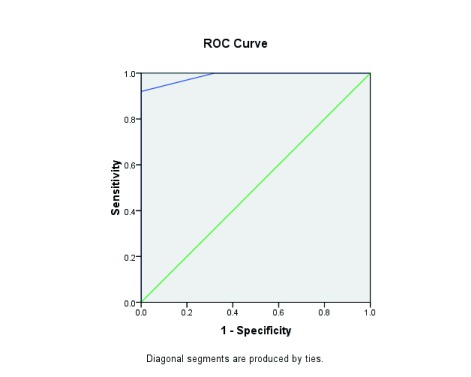
ROC analysis for sensitivity and specificity of mean platelet volume.

The MPV of patients with active-SLE (n=25, M=7.12, SD=1.01) was numerically lower than those in the inactive-SLE group (n=25, M= 10.12, SD=0.97). An independent sample t-test was run to compare the means of the two groups. The assumption of normality was tested by Kolmogorov-Samirnov test and was found tenable ( *P*> 0.05). Moreover, similar results were obtained on Skewness and Kurosis testing (skewness=0.01, kurtosis= -1.06). The assumption of homogeneity of variances was tested using Levene’s test and was found tenable (F (48) = 0.23; *P*= 0.63). The results of the independent t-test showed a statistically significant difference between the mean values of MPV of the two groups(t (48) = 10.69; *P*<0.001; Cohen’s D= 3.02). The 95% Confidence Interval (CI) was -3.56 to -2.44. Receiver operator characteristic (ROC) curve was used to check the specificity and sensitivity of MPV ( Figure 1). The ROC curve had an area under the curve of 0.98. At a value of 8.5fl for MPV, the sensitivity and specificity were 92% and 100%, respectively, ( *P*<0.001;95%CI -0.96 to +1.01). At a cutoff value of 8.5fl, MPV has a maximum sensitivity and specificity. Therefore, we recommend that, at an MPV value of <8.5fl, the probability of SLE increases remarkably.

The SLEDAI scores between the two groups, active-SLE (M=16.36, SD=4.48) and inactive-SLE (M=3, SD=0.82), varied at a statistically significant level of *P*<0.001. The ESR was higher in patients with active SLE (M=49.52, SD=12.93) than those with stable disease (M=13.76 SD=1.72)( *P*<0.001). The details of the different hematological parameters are given in Table 5.

**Table 5.** Comparison of different test variables between the two study groups. SLEDAI, Systemic Lupus Erythematosus Disease Activity; MPV, mean platelet volume; ESR, erythrocyte sedimentation rate; WBC, white blood cells; Hb, hemoglobin.

Variable	Active-SLE		Inactive-SLE		*P* value
	Mean	SD	Mean	SD	
SLEDAI	16.36	4.48	3.00	0.82	< 0.001
MPV (fl)	7.12	1.01	10.12	0.97	< 0.001
ESR (1 ^st^ hour)	49.52	12.93	13.76	1.72	< 0.001
Platelets×10 ^3^/mm ^3^	259.72	1.10	259.68	1.03	0.89
WBC/mm ^3^	5422.00	396.68	5896.00	79.53	< 0.001
Hb (gm/dl)	11.64	1.04	13.56	0.87	< 0.001

Pearson’s correlation test was run to assess any relationship between MPV, and ESR in the active-SLE group. The results showed a statistically significant, negative correlation of MPV with ESR (r= -0.93, *P*<0.001). The spearman’s rho correlation demonstrated that, MPV had a strong but inverse correlation with SLEDAI, (r _s_= -.89, *P*<0.001). However, it showed a strong but positive correlation between ESR and SLEDAI (r _s_=0.90, *P*<0.001). Hence, it can be argued that increased disease activity of SLE is associated with both a higher ESR and SLEDAI score, and a correspondingly low MPV ( *P*<0.001).

Raw data of disease severity indicators in lupus Click here for additional data file. Copyright: © 2017 Khan A et al. 2017 Data associated with the article are available under the terms of the Creative Commons Zero "No rights reserved" data waiver (CC0 1.0 Public domain dedication).

## Discussion

SLE, which is more common in Black women, has a female to male ratio of approximately 9:1 ^12^. Our study group comprised of 84% female and 16% male population. Furthermore, most of the participants in our study were in the third decade of life. Participants with active-SLE were younger than those with the stable form of the disease. These findings are comparable to international statistics ^18, 19^. We observed that MPV was significantly lower in patients with active lupus than those without a flare. Similarly, we found that, MPV had a tendency to be lower with a correspondingly higher ESR in individuals with actively flaring SLE and vice versa. Moreover, MPV had a significant inverse correlation with both SLEDAI and ESR. Thus, it can be argued that, as an indicator of disease activity in patients with SLE, MPV may be a breakthrough hematological biomarker to monitor disease activity along-with both ESR and SLEDAI-2000.

Gasparyan *et al.* concluded that high MPV correlated with a variety of diseases, like cardio- and cerebrovascular disorders, venous and arterial thrombosis and low-grade inflammatory conditions ^20^. However, it was observed that, high intensity inflammatory disorders, such as active rheumatoid arthritis or relapses of familial Mediterranean fever, had low values of MPV, which could be reverted with anti-inflammatory medications ^21– 23^. Although we did not check the effect of anti-inflammatory medications, like corticosteroids, on MPV, we observed a strong inverse relationship of MPV with lupus severity and activity. Therefore, we recommend MPV as one of the markers of disease activity in patients with SLE.

Apart from MPV, ESR has traditionally been used as a marker of disease severity in patients with inflammatory conditions, and SLE, specifically ^24^. Similarly, SLEDAI was promulgated in 1985 as a tool to assess disease activity in lupus. This was later modified in 2002 and re-introduced as SLEDAI-2000 ^25^. SLEDAI-2000 has been validated against classic SLEDAI and is an important predictor of prognosis and mortality ^26^. High disease activity will increase ESR and SLEDAI-2000 score ^24, 26^. Our study demonstrated that, MPV correlated inversely with both ESR and SLEDAI-2000. Thus it can be stated that, actively flaring lupus in a given patient will be characterized by a higher ESR and SLEDAI-2000 score and a correspondingly lower MPV.

Why is MPV low in active SLE? The answer cannot be clearly stated. However, previous studies have shown that, in active inflammatory conditions, especially rheumatoid arthritis, large and activated platelets are consumed preferentially at the site of inflammation, leaving small platelets behind ^21, 22^. This may also explain lower MPV values in actively flaring SLE patients in our study group as well as in other studies which demonstrated lower MPV values in patients with active lupus ^11^.

Considering active-SLE as a state of severe inflammation, those with active disease in our study were treated with 1g daily dose of methylprednisolone for three days, followed by a maintenance dose of 1mg/Kg oral prednisolone for another 4–6 weeks. Although, all participants with active lupus achieved dramatic symptomatic and clinically obvious improvement, MPV was not studied after the completion of steroid therapy. However, in other studies, where pre-treatment MPV was compared with post-treatment MPV, it was observed that, after successful treatment with anti-inflammatory medications, MPV reverted back to normal ^22– 27^. Considering this data, we would advocate future studies focusing on comparing pre- and post-treatment MPV in patients treated with corticosteroids for acute flare of SLE.

It is noteworthy that; although, most of the studies found an inverse relationship between active-SLE and MPV in adults, a positive association was observed between MPV and disease activity in juvenile lupus erythematosus ^10^. A similar observation was demonstrated in an Indian study by Sarkar RN *et al.* ^28^. These findings of positive correlation between MPV and disease activity in lupus are in sharp contrast to the results of our and similar previous studies ^8, 9^.

Notably, the results of this study were in accordance with the expectations of authors. Moreover, considering recent research studies showing a link between low MPV and disease activity in SLE patients, our study will add further evidence. However a limitation to the conduct and results of this study was a small sample size. This is because, SLE is not very common in Pakistan. Therefore, in order to highlight the actual role of MPV as a biomarker of lupus severity, we recommend that, cohort studies should be done in future, both in Pakistan and abroad.

## Conclusions

MPV is an excellent biomarker to assess disease activity in SLE, as higher disease activity will reduce MPV. Moreover, MPV has an inverse relationship with both ESR and SLEDAI. At a cutoff value of less than 8.5fl, MPV has an excellent sensitivity and specificity for determining disease activity in SLE.

## Ethics approval and consent

This study was approved by Ethics Review Committee of Khyber Teaching Hospital, Peshawar, Pakistan (approval number, KTH/2015/Med-A/86C). Informed written consent was obtained from every participant.

## Data availability

The data referenced by this article are under copyright with the following copyright statement: Copyright: © 2017 Khan A et al.

Data associated with the article are available under the terms of the Creative Commons Zero "No rights reserved" data waiver (CC0 1.0 Public domain dedication). 

**Dataset 1: Raw data of disease severity indicators in lupus.** This file contains data regarding disease severity indicators and demographics of patients with SLE. This coded data was stored on SPSS version 16. Group: 1, active-SLE; 2, inactive-SLE. Gender: 1, male; 2, female. SLEDAI, systemic lupus erythematosus disease activity index; MPV, mean platelet volume; ESR, erythrocyte sedimentation rate; WBC, white blood cell (thousand/mm ^3^); Hb, hemoglobin (gm/dl); platelets, platelet count × 10 ^3^. doi, 10.5256/f1000research.10763.d151064 ^29^

## Supplementary material

**Supplementary File 1: Lupus severity and disease activity questionnaire.** This questionnaire was used to gather data from the participants. SLEDAI, systemic lupus erythematosus disease activity index; MPV, mean platelet volume; ESR, erythrocyte sedimentation rate; WBC, white blood cell (thousand/mm ^3^); active SLE, SLEDAI >5 points; inactive SLE, SLEDAI <5 points.
