# Supplementary material for: Mean Platelet Volume (MPV) as an indicator of disease activity and severity in lupus
Source: F1000Res. 2017 Mar 16;6:126. Originally published 2017 Feb 10. [Version 3] doi: 10.12688/f1000research.10763.3 (PMC5365216; doi:10.12688/f1000research.10763.3)
Supplement: Supplementary file 2 [file f1000research-6-11985-s0001.tgz › 74599159-23a6-42ab-871f-7b12576bb4ac.docx]

**Lupus severity and disease activity questionnaire**

A: **Demography**

1. Serial number:____________________
2. Patient ID:______________________
3. Age:_________________
4. Gender: 1) Male 2) Female
5. Ethnicity: 1) Pakistani 2) Afghan 3) Other
6. Occupation: 1) Works outdoors (e.g. farmer, laborer, driver) 2) Works indoors (e.g. doctor, banker, house worker)
7. Time since diagnosis (months):__________

B: **Disease Activity**

1. SLEDAI score:______________

**C) Classification of lupus**

1. Active SLE
2. Inactive SLE

**D)** **Hematological parameters**

1. ESR
2. MPV
3. Hemoglobin (gm/dl)
4. Platelets (count/mm^3)^:
5. WBC (count/mm^3)^

**E) Renal parameters**

1. Creatinine (mg/dl)
2. Urea (mg/dl)
3. Daily urinary potein (gm/day)____________

**F) Renal histology** (in patients with >0.5gm urinary protein/day) ___________________

**G) Clinical features**

- **Pregnancy (currently)**

1. Yes
2. No

- **Recurrence of SLE**

1. Yes
2. No

- If ‘yes’ to above, how many recurrences (please specify) __________
- **Tick the clinical feature that is present;**

1. Arthritis
2. Malar rash
3. Photosensitivity
4. Patchy alopecia
5. Pleurisy
6. Pericarditis
7. Neuropsychiatric problems
8. Oral ulcers
9. Anti-dsDNA antibodies
10. Antinuclear factor
11. Proteinuria (>0.5gm/day)
12. Non-specific aches and pains
13. Malaise
14. Others (please specify) _______________________________________________
