# Supplementary material for: Mean Platelet Volume (MPV) as an indicator of  disease activity and severity in lupus
Source: F1000Res. 2017 Mar 16;6:126. Originally published 2017 Feb 10. [Version 3] doi: 10.12688/f1000research.10763.3 (PMC5365216; doi:10.12688/f1000research.10763.3)
Supplement: Peer review history

# Peer review: Referee response for version 3

**DOI:** 10.5256/f1000research.11985.r21027

**Published:** 2017-03-20

**Authors:** Guillermo Delgado-García

**Affiliations:** 1 National Institute of Neurology and Neurosurgery, Mexico City, Mexico

It has been a pleasure for me to review your valuable work. I applaud again your academic openness and your critical spirit. I want to add the following:

 5. This point was appropriately addressed.

 8. This point was appropriately addressed.

 10. This point was appropriately addressed.

 12. This point was addressed.

I have read this submission. I believe that I have an appropriate level of expertise to confirm that it is of an acceptable scientific standard.

# Peer review: Referee response for version 2

**DOI:** 10.5256/f1000research.11885.r20507

**Published:** 2017-03-14

**Authors:** Alina Dima

**Affiliations:** 1 Department of Clinical Epidemiology, Aarhus University Hospital, Aarhus, Denmark

Dear authors,

 Thank you for all changes made.

 Best regards,

 AD

I have read this submission. I believe that I have an appropriate level of expertise to confirm that it is of an acceptable scientific standard.

# Peer review: Referee response for version 2

**DOI:** 10.5256/f1000research.11885.r20506

**Published:** 2017-02-28

**Authors:** Guillermo Delgado-García

**Affiliations:** 1 National Institute of Neurology and Neurosurgery, Mexico City, Mexico

First of all I want to thank Abidullah Khan (Pakistan) for his punctual answers, and in the same way I take this opportunity to applaud his openness to academic debate. I want to add the following:

1. This point was appropriately addressed.
2. This point was appropriately addressed.
3. This point was appropriately addressed. However, I am not sure if we are falling into a conflict of interest by including an article written by the reviewer under the suggestion of the same reviewer. If possible, I would like to leave this decision to another reviewer in order to avoid any conflict of interest.
4. This point was appropriately addressed.
5. Taking into account that you also used a formula to determine the appropriate sample size for detecting a difference between the means of two samples, I think it would be good to cite the previous report on which this calculation was based (i.e., the article from where you got the values to make your sample size estimation). Likewise, I think it would be good to know how much is "more than 0.8" (power).
6. This point was appropriately addressed.
7. I agree with your proposal, so lets leave them in two separate tables.
8. The final SLEDAI score is an ordinal variable ^1^, so I consider that it would be more appropriate to evaluate the correlation between this variable and MPV using Spearman's rho.
9. This point was appropriately addressed.
10. I do not know if you are close to the word limit. If this is not the case, perhaps it would also be worth mentioning the studies in adults ^2^ that yielded results similar to the study in patients with juvenile lupus ^3^.
11. This point was appropriately addressed.

An additional final comment:

      12.The following lines in the Discussion seem particularly bold to me, perhaps it would be worth rephrasing them:

 a) "Thus, it can be argued that, as an indicator of disease activity in patients with SLE, MPV was **as effective** as both ESR and SLEDAI-2000".

 b) "Therefore, we recommend MPV as a global marker of disease activity in patients with SLE".

I have read this submission. I believe that I have an appropriate level of expertise to confirm that it is of an acceptable scientific standard, however I have significant reservations, as outlined above.

## References

[1] LewRALiangMHDorosG: Statistical considerations for stopping systemic lupus erythematosus clinical trials earlier. *Arthritis Res Ther*.2015;17: 10.1186/s13075-015-0874-0 345 10.1186/s13075-015-0874-0 26634344PMC4669672

[2] SarkarRNBhattacharyaKBanerjeeM: Mean platelet volume as an indicator of disease activity in systemic lupus erythematosus. *J Assoc Physicians India*.2016;64(1) :68-69 27727907

[3] YavuzSEceA: Mean platelet volume as an indicator of disease activity in juvenile SLE. *Clin Rheumatol*.2014;33(5) : 10.1007/s10067-014-2540-3 637-41 10.1007/s10067-014-2540-3 24567240

# Response

**Published:** 2017-03-06

**Authors:** Abidullah Khan

**Affiliations:** Khyber Teaching Hospital/ Khyber Medical College, Peshawar, Pakistan

Thank you again very much for your precious time and tremendous energy in revisiting our manuscript critically. We have made the following changes in response to your remaining invaluable reservations. 

**5: **Taking into account that you also used a formula to determine the appropriate sample size for detecting a difference between the means of two samples, I think it would be good to cite the previous report on which this calculation was based (i.e., the article from where you got the values to make your sample size estimation). Likewise, I think it would be good to know how much is "more than 0.8" (power).

**Reply:** This has been amended as advised. 

**8: **The final SLEDAI score is an ordinal variable ^1^, so I consider that it would be more appropriate to evaluate the correlation between this variable and MPV using Spearman's rho.

**Reply: **Spearman's rho has been used to assess any relationship of MPV and ESR with SLEDAI. However, the relation of MPV with ESR has been assessed through Pearson's correlation test.

**10:** The Indian study has now been referred to. The study by Yavuz S et al had already been cited.

**Additional comments:** The final two sentences pointed to (a and b), have been rephrased.

 I hope it works this time. Thanks again.

# Peer review: Referee response for version 1

**DOI:** 10.5256/f1000research.11605.r20320

**Published:** 2017-02-17

**Authors:** Alina Dima

**Affiliations:** 1 Department of Clinical Epidemiology, Aarhus University Hospital, Aarhus, Denmark

1. **Title**: I don’t find the term “monitoring” appropriate as it is presented a cross-sectional study with only one MPV determination and I so think that the use of assessment or determination could be tried.
  Also the term MPV (as possible marker of SLE disease activity) should be included in title.
2. **Abstract**: First two phrases of the abstract are too general, not directly related to this article subject. I think that for the background the idea MPV – activity – Inflammation – SLE is enough.
  In the method we understand that 25 patients with active vs. 25 inactive were chosen ... it should be clear noted; e.g. cross-sectional, prospective, successively inclusion and then how the two subgroups were defined.
  I don’t think that the details of statistic should appear in the abstract - methods, maybe a more detailed conclusion.
3. ** Introduction**: As for the abstract, I find the first two paragraphs are too general, not directly related to the article topic.
  For the classification criteria, instead of classification criteria up-dated ACR 1997 (that should be cited properly) I would propose the use of SLICC 2012 or both criteria sets; but anyway, the diagnosis would be the same as the last criteria set is more sensitive.
4. **Methods/ Results**: There are many exclusion criteria presented, maybe there could be some words in introduction or discussion about these factors influence on MPV (as it was discussed for thrombocytopenia) and reasons of exclusion.
  I understand from methods that it was a cross-sectional study with random, consecutive inclusion and then the lot was split in two groups according to SLE disease activity (assessed by SLEDAI).
  How was the SLEDAI cut-off of 5 points determined? This cut-off appears in previous published research, it is the mean value (as I understand all variables turned out to be parametric), or the group was split in two groups of similar patients number?
  And then, the bivariate correlations were realized only in the group with active SLE, so the results are applicable only in this subgroup of patients.
  I would propose a logistic regression for the entire lot (50 patients), with MPV, ESR (and adjusted for age and gender) as predictors for high SLEDAI.
  However, the degree of correlation obtained is very high and so it sustains the research conclusion.
5. **Discussion**: the phrase “SLEDAI was as effective as both ESR and MPV” should be written differently (MPV was correlated with/ is an effective marker of ...) as the research is about MPV, disease activity was assessed by SLEDAI and ESR was noted as parameter known to correlates with disease activity.
  In the third paragraph, I don’t find a relation between the discussion on CRP and the same paragraph last phrase. Of course, data on ESR usefulness as marker of SLE disease activity should be discussed in order to understand why SLEDAI and ESR as standard. And maybe also data on ESR and MPV in lupus or other diseases, if there are.
6. **Conclusion**: I think that the first phrase of conclusion should be reformulated. The term “monitor” I already discussed; “higher disease will reduce MPV” could be more specific; and “vice versa” I’m not sure that this can be concluded as the study does not discuss distinctly the relation between higher MPV with lower activity in the subgroup of inactive lupus patients (of course, it looks logical).
  I think also that the present last phrase of conclusion should be ended “ sensitivity and specificity for ...” and in active SLE, without APS ...

I have read this submission. I believe that I have an appropriate level of expertise to confirm that it is of an acceptable scientific standard, however I have significant reservations, as outlined above.

# Response

**Published:** 2017-02-21

**Authors:** Abidullah Khan

**Affiliations:** Khyber Teaching Hospital/ Khyber Medical College, Peshawar, Pakistan

We are really grateful to Alina Dima for her invaluable comments on our article. Please find below a point-wise response to the comments.

1. **Title**: "I don’t find the term “monitoring” appropriate as it is presented a cross-sectional study with only one MPV determination and I so think that the use of assessment or determination could be tried. Also the term MPV (as possible marker of SLE disease activity) should be included in title."
***Reply: This suggestion has been incorporated.***
2. **Abstract**: "First two phrases of the abstract are too general, not directly related to this article subject. I think that for the background the idea MPV – activity – Inflammation – SLE is enough. In the method we understand that 25 patients with active vs. 25 inactive were chosen ... it should be clear noted; e.g. cross-sectional, prospective, successively inclusion and then how the two subgroups were defined. I don’t think that the details of statistic should appear in the abstract - methods, maybe a more detailed conclusion."
***Reply: Abstract has been re-phrased as suggested. However, we believe that, the statistics should appear in the abstract, as they give an overall idea to the researcher of what we did and what s/he may expect to find in the results?***
3. **Introduction**: As for the abstract, I find the first two paragraphs are too general, not directly related to the article topic. For the classification criteria, instead of classification criteria up-dated ACR 1997 (that should be cited properly) I would propose the use of SLICC 2012 or both criteria sets; but anyway, the diagnosis would be the same as the last criteria set is more sensitive.
***Reply: We have intentionally left the first two paragraphs as general looking. This is for the purpose of education of the young researchers and to give an impression as, how does SLE behave normally ?***
4. **Methods/ Results**: There are many exclusion criteria presented, maybe there could be some words in introduction or discussion about these factors influence on MPV (as it was discussed for thrombocytopenia) and reasons of exclusion.
***Reply: Sentence has now been added regarding the influence of the set exclusion criteria on MPV and the study results.***
  I understand from methods that it was a cross-sectional study with random, consecutive inclusion and then the lot was split in two groups according to SLE disease activity (assessed by SLEDAI). How was the SLEDAI cut-off of 5 points determined? This cut-off appears in previous published research, it is the mean value (as I understand all variables turned out to be parametric), or the group was split in two groups of similar patients number? And then, the bivariate correlations were realized only in the group with active SLE, so the results are applicable only in this subgroup of patients. I would propose a logistic regression for the entire lot (50 patients), with MPV, ESR (and adjusted for age and gender) as predictors for high SLEDAI. However, the degree of correlation obtained is very high and so it sustains the research conclusion.
***Reply: The recommended changes have been made.***
5. **Discussion**: the phrase “SLEDAI was as effective as both ESR and MPV” should be written differently (MPV was correlated with/ is an effective marker of ...) as the research is about MPV, disease activity was assessed by SLEDAI and ESR was noted as parameter known to correlates with disease activity. In the third paragraph, I don’t find a relation between the discussion on CRP and the same paragraph last phrase. Of course, data on ESR usefulness as marker of SLE disease activity should be discussed in order to understand why SLEDAI and ESR as standard. And maybe also data on ESR and MPV in lupus or other diseases, if there are.
***Reply: Discussion has been modified at points suggested by the respectable reviewer.***
6. **Conclusion**: I think that the first phrase of conclusion should be reformulated. The term “monitor” I already discussed; “higher disease will reduce MPV” could be more specific; and “vice versa” I’m not sure that this can be concluded as the study does not discuss distinctly the relation between higher MPV with lower activity in the subgroup of inactive lupus patients (of course, it looks logical). I think also that the present last phrase of conclusion should be ended “ sensitivity and specificity for ...” and in active SLE, without APS ...
***Reply: The conclusion has been amended as recommended by the reviewer. Moreover, the term 'vice versa'  has been removed.***

Thank you once more for your time and precious comments.

# Peer review: Referee response for version 1

**DOI:** 10.5256/f1000research.11605.r20146

**Published:** 2017-02-13

**Authors:** Guillermo Delgado-García

**Affiliations:** 1 National Institute of Neurology and Neurosurgery, Mexico City, Mexico

In this study, Khan et al explored the relationship between SLE disease activity and mean platelet volume (MPV). After reading this article, I have the following suggestions:

1. I think it would be good if the title were more specific (perhaps including such terms as “platelet size” or “platelet volume”).
2. Contrary to what is mentioned in the last paragraph of the Introduction, Rupa-Matysek et al. ^1^ did not include SLE patients in their study.
3. Our team also published an article on this topic ^2^, which is not mentioned in the Introduction, even when it appeared online before that of Yolbas et al ^3^.
4. I would like to know if the sampling technique was consecutive or random. I'm inclined to think that consecutive (i.e., non-random) sampling was used.
5. I'm not sure if this STEPS Sample Size Calculator was the ideal one to estimate the sample size in the present study. I would like to know what other values were used in this calculation (level of confidence measure, MOE, baseline levels of the indicators, Deff, etc). I would also like to know which outcome was used to estimate the sample size. MPV? If so, why was not used a formula to determine the appropriate sample size for detecting a difference between the means of two samples?
6. One merit of this study when comparing it with others is that all the blood samples were analyzed within less than one hour after sampling, since MPV increases over time in EDTA tubes.
7. I think that the bar chart is not ideal to display demographic information. Perhaps one table could be just enough.
8. I suggest the first two tables could be merged into one.
9. When calculating the correlations it is important to remember that the SLEDAI is an ordinal variable ^4^.
10. In the discussion, when addressing the issue of pathophysiological mechanisms that could explain the decrease in MPV, two articles are cited. However, contrary to what is mentioned, neither of these articles deals with SLE patients.
11. It would be worthwhile to further discuss the findings of this study by comparing them more specifically with the other studies on this same topic.
12. There are some grammatical errors that would be worth correcting (e.g., an unnecessary semicolon in the second paragraph of the Methods).

I have read this submission. I believe that I have an appropriate level of expertise to confirm that it is of an acceptable scientific standard, however I have significant reservations, as outlined above.

## References

[1] Rupa-MatysekJGilLWojtasińskaECiepłuchKLewandowskaMKomarnickiM: The relationship between mean platelet volume and thrombosis recurrence in patients diagnosed with antiphospholipid syndrome. *Rheumatol Int*.2014;34(11) : 10.1007/s00296-014-2996-0 1599-605 10.1007/s00296-014-2996-0 24671503PMC4209097

[2] Delgado-GarcíaGGalarza-DelgadoDÁColunga-PedrazaIBorjas-AlmaguerODMandujano-CruzIBenavides-SalgadoDMartínez-GranadosRJAtilano-DíazA: Mean platelet volume is decreased in adults with active lupus disease. *Rev Bras Reumatol Engl Ed*.56(6) : 10.1016/j.rbre.2016.03.003 504-508 10.1016/j.rbre.2016.03.003 27914597

[3] YolbasSYildirimAGozelNUzBKocaSS: Hematological Indices May Be Useful in the Diagnosis of Systemic Lupus Erythematosus and in Determining Disease Activity in Behçet's Disease. *Med Princ Pract*.2016;25(6) : 10.1159/000447948 510-516 10.1159/000447948 27348861PMC5588503

[4] LewRALiangMHDorosG: Statistical considerations for stopping systemic lupus erythematosus clinical trials earlier. *Arthritis Res Ther*.2015;17: 10.1186/s13075-015-0874-0 345 10.1186/s13075-015-0874-0 26634344PMC4669672

# Response

**Published:** 2017-02-21

**Authors:** Abidullah Khan

**Affiliations:** Khyber Teaching Hospital/ Khyber Medical College, Peshawar, Pakistan

We thank Guillermo Delgado-Garcia from Mexico for reading our manuscript thoroughly and for pin-pointing various discrepancies. We have now corrected the shortcomings and we believe that, the changes made in the light of recommendation of our honorable reviewer will add a lot to the science of our article. Please find below, a point-by-point response to the reviewers comments;

1. The title of the article has been modified as suggested.
2. **" *Contrary to what is mentioned in the last paragraph of the Introduction, Rupa-Matysek et al. did not include SLE patients in their study".***
  This has been rephrased. Thank you for identifying this.
3. The article published by your team is very informative and has now been referred to.
4. The sampling technique was consecutive. The sentence has been rewritten. Sorry for the mistake in version 1.
5. ***"I'm not sure if this STEPS Sample Size Calculator was the ideal one to estimate the sample size in the present study. I would like to know what other values were used in this calculation (level of confidence measure, MOE, baseline levels of the indicators, Deff, etc). I would also like to know which outcome was used to estimate the sample size. MPV? If so, why was not used a formula to determine the appropriate sample size for detecting a difference between the means of two samples?"***
  We used both the methods. However, we mentioned the WHO sample size calculator only. All the missing info has now been added. 
6. Thanks for your appreciation of the merits of our study.
7. Bar chart has now been replaced with a table as suggested.
8. ***"I suggest the first two tables could be merged into one". ***
  Merging the two tables will make the tables look lengthy and possibly confusing. Therefore, we believe that, illustration of the clinical features of the two groups in separate tables will be better read and understood.
9. " ***When calculating the correlations it is important to remember that the SLEDAI is an ordinal variable." ***
  We understand that SLEDAI is an ordinal variable. However, we used the final SLEDAI score rather than the overall conclusion (active/inactive) while running the correlation tests. As the SLEDAI score is continuous and in our case, was normally distributed, we believe running a correlation test was tenable.
10. " ***In the discussion, when addressing the issue of pathophysiological mechanisms that could explain the decrease in MPV, two articles are cited. However, contrary to what is mentioned, neither of these articles deals with SLE patients." ***
  This area has been rephrased. Thank you for pointing it out.
11. In our opinion, discussing the study further, will unnecessary lengthen the text of this article.
12. Every effort has been made to correct the English and grammar.
  We thank you once more for your efforts.
